# Adsorbents Reduce Aflatoxin M_1_ Residue in Milk of Healthy Dairy Cow Exposed to Moderate Level Aflatoxin B_1_ in Diet and Its Exposure Risk for Humans

**DOI:** 10.3390/toxins13090665

**Published:** 2021-09-17

**Authors:** Manqian Cha, Erdan Wang, Yangyi Hao, Shoukun Ji, Shuai Huang, Lihong Zhao, Wei Wang, Wei Shao, Yajing Wang, Shengli Li

**Affiliations:** 1 College of Animal Science, Xinjiang Agricultural University, Urumqi 830052, China; chamanqian@163.com (M.C.); dksw@xjau.edu.cn (W.S.); 2 State Key Laboratory of Animal Nutrition, College of Animal Science and Technology, China Agricultural University, Beijing 100193, China; wangerdan@cau.edu.cn (E.W.); B20193040338@cau.edu.cn (Y.H.); huangshuai@hainanu.edu.cn (S.H.); zhaolihongcau@cau.edu.cn (L.Z.); wei.wang@cau.edu.cn (W.W.); yajingwang@cau.edu.cn (Y.W.); 3 College of Animal Science, Hebei Agricultural University, Baoding 071000, China; dkjsk@hebau.edu.cn

**Keywords:** aflatoxin B_1_, moderate risk level, adsorbents, aflatoxin M_1_, transfer rate, dairy cows, exposure risk assessment

## Abstract

This study investigated the effect of moderate risk level (8 µg/kg) AFB_1_ in diet supplemented with or without adsorbents on lactation performance, serum parameters, milk AFM_1_ content of healthy lactating cows and the AFM_1_ residue exposure risk in different human age groups. Forty late healthy lactating Holstein cows (270 ± 22 d in milk; daily milk yield 21 ± 3.1 kg/d) were randomly assigned to four treatments: control diet without AFB_1_ and adsorbents (CON), CON with 8 μg/kg AFB_1_ (dry matter basis, AF), AF + 15 g/d adsorbent 1 (AD1), AF + 15 g/d adsorbent 2 (AD2). The experiment lasted for 19 days, including an AFB_1_-challenge phase (day 1 to 14) and an AFB_1_-withdraw phase (day 15 to 19). Results showed that both AFB_1_ and adsorbents treatments had no significant effects on the DMI, milk yield, 3.5% FCM yield, milk components and serum parameters. Compared with the AF, AD1 and AD2 had significantly lower milk AFM_1_ concentrations (93 ng/L vs. 46 ng/L vs. 51 ng/L) and transfer rates of dietary AFB_1_ into milk AFM_1_ (1.16% vs. 0.57% vs. 0.63%) (*p* < 0.05). Children aged 2–4 years old had the highest exposure risk to AFM_1_ in milk in AF, with an EDI of 1.02 ng/kg bw/day and a HI of 5.11 (HI > 1 indicates a potential risk for liver cancer). Both AD1 and AD2 had obviously reductions in EDI and HI for all population groups, whereas, the EDI (≥0.25 ng/kg bw/day) and HI (≥1.23) of children aged 2–11 years old were still higher than the suggested tolerable daily intake (TDI) of 0.20 ng/kg bw/day and 1.00 (HI). In conclusion, moderate risk level AFB_1_ in the diet of healthy lactating cows could cause a public health hazard and adding adsorbents in the dairy diet is an effective measure to remit AFM_1_ residue in milk and its exposure risk for humans.

## 1. Introduction

Aflatoxins are a group of toxic secondary metabolites mainly produced by several species of the genus and contaminate animal feeds and products [1,2]. Among approximately 18 identified aflatoxins [3], aflatoxin B_1_ (AFB_1_) has been the most widely studied and problematic mycotoxin in dairy cows [4]. AFB_1_ in the dairy diet is partly bio-transformed into aflatoxin M_1_ (AFM_1_) in the liver and then be secreted into the milk [5,6] and further contaminate dairy products, such as fresh milk, cheese, ice cream, powdered milk, yogurt, and baby formula. While acute exposure to a high dose of AFM_1_ can result in vomiting, abdominal pain and even death, chronic exposure to low doses of AFM_1_ may lead to liver cancer [7,8], posing a significant human health hazard [9,10]. In particular, children aged 2–4 years old had the highest risk of exposure to AFM_1_ in milk [11]. Therefore, AFM_1_ in milk need to be monitored and AFB_1_ in dairy feed should be limited at the lowest possible levels.

More than 60 countries have set up strict guidelines for maximum residue level (MRL) of AFM_1_ in milk [12] and more than 100 countries have issued specific regulated or recommended limits for mycotoxin control in products intended for animal feeds [13]. The MRL of AFM_1_ in raw milk is 0.5 µg /L in the United States and China [14,15], while the European Union set the level at 0.05 µg /L [16]. The maximum permissible amount of AFB_1_ in dairy feed has also been established, ranging from 20 µg/kg in the United States to 10 µg/kg in China and 5 µg/kg in the European Union [17]. However, these legal regulations have not eradicated milk AFM_1_ successfully [3,18,19]. The estimated daily intake (EDI) of milk AFM_1_ in previous studies was reported to exceed the tolerable daily intake (TDI) limit of 0.20 ng/kg bw/day in several countries [11,20,21,22,23].

Based on field experience and laboratory investigation, we defined the dietary AFB_1_ risk of dairy cows into four levels: critical (>20 µg/kg), high (10–20 µg/kg), moderate (5–10 µg/kg) and low (<5 µg/kg). Many surveys about AFB_1_ contamination in feedstuffs have been done worldwide, the overall data showed that most of the AFB_1_ risk in the dairy feed is low and moderate levels [13,24,25,26]. Biomin Inc. (Ferndale, MI, USA) conducted a worldwide survey of mycotoxin contamination in feed ingredients in 2018 [27] and 2019 [28], the results showed that the aflatoxin positive rates of finished feed in Asia were 44% and 30%, respectively, and the median of positive samples were both 8 µg/kg [29]. Meanwhile, most researchers conducted the field trails in a critical high level of (>20 µg/kg) AFB_1_ dosages from 20 µg/kg [19,30,31], 22.28 µg/kg (naturally contaminated) [32], 40 µg/kg [31], 63 µg/kg [17], 76 µg/kg (1725 µg/d) [33], 100 µg/kg [34,35], 120 µg/kg [36] and up to 300 µg/kg in diet to investigate their negative effects to the cows [34]. Furthermore, there were no field trials that estimated the human exposure risk to milk AFM_1_ residue before. Although previous epidemiological surveys on AFM_1_ in commercial and raw milk have assessed their exposure risk for humans, data on AFB_1_ content in the diets were usually unusable. Meanwhile, when the prevention of aflatoxin contamination with crops and grains during pre-harvest and storage fails, adding AFB_1_ adsorbents to dairy diets was proved to be a very effective option to mitigate the negative impact of AFB_1_ [29]. However, few studies have determined the effects of moderate risk AFB_1_ and adsorbents in the diet on production performance and milk AFM_1_ concentration of lactating dairy cows and the risk assessment of milk AFM_1_ residual for different populations, although most of the dairy cows are likely facing moderate risk AFB_1_ exposure every day.

Thus, in the present study, we collaborated with the European Horizon Project, set up a moderate risk level (8 µg/kg) of AFB_1_ to fill up the MRL gap between China and European Union. The objectives of this study were to investigate the effects of supplemental moderate risk level AFB_1_ and two adsorbents on lactation performance, serum parameters, milk AFM_1_ content of dairy cows and estimate human exposure risk to current milk AFM_1_ residue.

## 2. Results

### 2.1. Feed Intake and Lactation Performance

All the cows in the four dietary treatments behaved normally and there were not any clinical signs of aflatoxicosis observed throughout the entire feeding trail. Meanwhile, there were no significant differences in the DMI, milk yield, 3.5% FCM yield and milk components (milk fat, protein, lactose and somatic cell count (SCC)) of the dairy cows fed with the moderate risk level of (8 μg/kg of diet dry matter) AFB_1_ with or without adsorbents, as shown in Table 1.

### 2.2. Serum Parameters

The effects of moderate risk level (8 µg/kg) of AFB_1_ with or without adsorbents on serum metabolite parameters are shown in Table 2. No significant difference was observed in the parameters of energy metabolism (GLU, NEFA and BHBA), liver function (ALT, AST and TP), oxidative stress (SOD, GSH-Px, TAOC and MDA) and gastrointestinal permeability (DAO, D-LA and LPS) (*p* > 0.05).

### 2.3. AFM_1_ Content in Milk

The effect of the moderate risk level of AFB_1_ with or without adsorbents on milk AFM_1_ content of dairy cows at steady state (day 7 to 14) is shown in Table 3. The AFM_1_ content in CON was below the detection limits (10 ng/L). The average AFM_1_ concentration in milk at the platform was 93 ng/L in the AF treatment, which was below the level of AFM_1_ MRL set by the United States and China, but it was 1.86 times higher than the legal limit of the European Union. The transfer rate of AFB_1_ from the diet into AFM_1_ in milk was 1.16% in the AF treatment. Compared to AF, AD1 and AD2 had significantly lower AFM_1_ concentrations in milk (93 ng/L vs. 46 ng/L vs. 51 ng/L), AFM_1_ excretion (1.94 μg/d vs. 0.96 μg/d vs. 1.06 μg/d) and transfer rate (1.16% vs. 0.57% vs. 0.63%) (*p* < 0.05). Meanwhile, AD1 had a greater reduction in AFM_1_ concentration (50.54% vs. 45.16%), AFM_1_ excretion (50.52% vs. 45.36%) and the transfer rate (50.86% vs. 45.69%) compared to AD2, but not statistically significant (*p* > 0.05).

**Figure 1:**
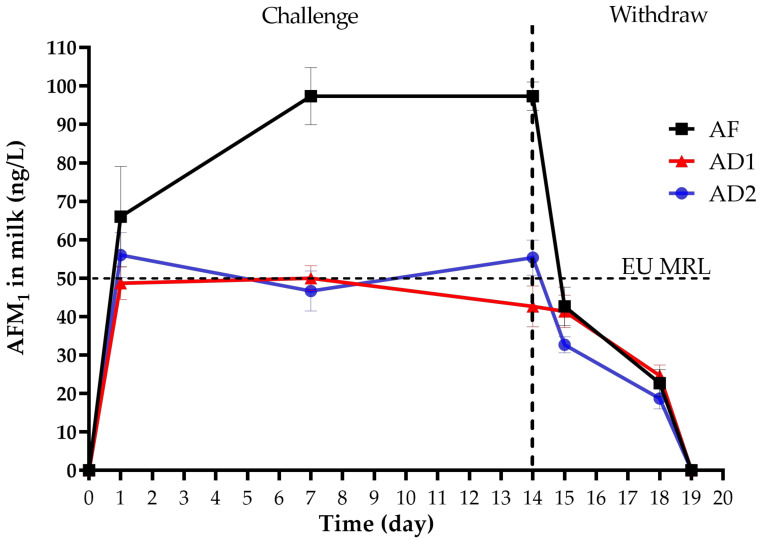
Effects of the moderate risk level of AFB_1_ with or without adsorbents on milk AFM_1_ concentration of dairy cows. AFB_1−_ challenge period: day 1 to 14; AFB_1−_ withdraw period: day 15 to 19. AF: the basal diet + 8 μg/kg AFB_1_; AD1: AF + 15 g/d adsorbent 1; AD2: AF + 15 g/d adsorbent 2. EU MRL: maximum residue level (MRL) of the European Union (50 ng/L).

The milk AFM_1_ concentrations in AF, AD1 and AD2 treatments throughout the entire experimental period are shown in Figure 1. The milk AFM_1_ concentrations of AF, AD1 and AD2 treatments reached a mean of 66, 49 and 56 ng/kg at 24 h after the first AFB_1_ administration, then they were maintained up to a relatively stable level at day 7 (93, 50 and 47 ng/L) and day 14 (93, 43 and 55 ng/L). Therefore, the steady-state (day 7–14) was defined with the average milk AFM_1_ concentrations of 93, 46 and 51 ng/L, respectively, in AF, AD1 and AD2 treatments. The milk AFM_1_ concentrations dropped to 43, 41 and 33 ng/L at 24 h after withdrawal AFB_1_ (day 15), continued decreasing in the following days and were undetectable on 5 days after withdrawing AFB_1_ administration (day 19).

Comparison of the transfer rate under different risk levels of dietary AFB_1_ with or without adsorbents or other detoxification agents in previous studies and the present study is shown in Table 4. In previous studies, offering detoxification agents to dairy cows challenged with different AFB_1_ dosages in the diet has shown a reduction in the transfer rate of AFB_1_ from diet to AFM_1_ in milk regarding production variables.

### 2.4. Exposure Risk Assessment

Based on the average AFM_1_ concentrations of milk in this study, the risk assessment of AFM_1_ exposure in different populations is calculated and shown in Table 5. It can be seen that EDI values for AF, AD1 and AD2 ranged from 0.17 to 1.02, 0.08 to 0.51 and 0.09 to 0.56 ng/kg bw/day, respectively, in different human age groups. HI values for AF, AD1 and AD2 ranged from 0.84 to 5.11, 0.41 to 2.52 and 0.46 to 2.80, respectively. Compared to AF, both AD1 and AD2 had reductions in EDI and HI in all age groups, whereas, the EDI (≥0.25 ng/kg bw/day) and HI (≥1.23) of children aged 2–11 years old were still higher than the TDI and 1.00 (HI). It is worth noting that the risk of AFM_1_ exposure was highest in milk consumers aged 2–4 years old, with an EDI of 1.02, 0.51 and 0.56 ng/kg bw/day and a HI of 5.11, 2.52 and 2.80 in AF, AD1 and AD2, respectively. Meanwhile, milk consumers aged 30–45 years old were found to have the lowest risk of AFM_1_ exposure, with an EDI of 0.17, 0.08 and 0.09 ng/kg bw/day and a HI of 0.84, 0.41 and 0.46 in AF, AD1 and AD2, respectively.

## 3. Discussion

All the cows in the four dietary treatments were in apparently healthy condition and there were not any clinical signs of aflatoxicosis observed throughout the entire feeding trail. The previous studies indicated that critical level of (≥20 µg/kg) AFB_1_: 20 µg/kg [2,19], 40µg/kg [2], 63 µg/kg (1197 µg/d) [17], 75 µg/kg (1725 µg/d) [33], 100 µg/kg [41], 117 µg/kg [37], 112 µg/kg [38] and adsorbents administration in diet had no significant effects on the production performance of dairy cows. However, Queiroz et al. [43] and Malinee et al. [32] reported that cows were exposed to naturally contaminated diets containing 22.28 µg/kg AFB_1_ resulted in a significant reduction of milk protein concentration and milk fat yield. It is noteworthy that the cows were exposed to naturally contaminated TMR diets may co-exposure to the mycotoxin combinations, which led to more adverse effects on the cows than purified AFB_1_.

The moderate risk level of (8 µg/kg) AFB_1_ with or without adsorbents in diet did not affect the GLU, NEFA and BHBA in the serum, indicating that moderate risk level AFB_1_ did not affect the energy metabolism of lactating dairy cows. It is well known that the liver is the main organ for AFB_1_ metabolism and the target organ for aflatoxicosis. While alanine aminotransferase (ALT), aspartate aminotransferase (AST) and total protein (TP) are the main liver function parameters of dairy cows, there were no statistically significant differences in ALT, AST and TP content among each dietary treatment in the current study. Likewise, both short-term addition of critical risk level (63 µg/kg) and long-term addition of critical risk level (20 µg/kg) AFB_1_ in the diet of cows did not cause statistically significant changes in ALT, AST and TP [17,19]. Meanwhile, Keller et al. (2015) reported that the yeast cell wall extracts and sodium alginate in adsorbent might stimulate both nonspecific and specific immunological responses, thus improving the performance of cows [51]. Further study is needed to understand the interaction between liver function parameters and AFB_1_ challenge to the healthy dairy cows. Xiong et al. reported that long-term critical high level (20 µg/kg) AFB_1_ significantly decreased serum concentrations of SOD, GSH-Px and TAOC of cows, meanwhile increased the serum MDA concentration [19]. In addition, dietary addition of vitamin E, yeast extract and sodium montmorillonite could alleviate oxidative stress in cows with the AFB_1_ challenge [2]. Diamine oxidase (DAO), D-lactic acid (D-LA) and Lipopolysaccharide (LPS) are the key indicators for the barrier function of the gastrointestinal mucosa, reflects the integrity of the intestinal mechanical barrier and the degree of damage [52,53]. The present study was consistent with previous results [2,19], cows that consumed diet contaminated with AFB_1_ did not affect their gastrointestinal permeability. In summary, moderate risk level (8 µg/kg) AFB_1_ with or without adsorbents in diet do not affect energy metabolism, liver function, oxidative stress and gastrointestinal permeability of the healthy dairy cows.

The aflatoxin B_1_, B_2_, G_1_, G_2_, deoxynivalenol, T-2 toxin, zearalenone and ochratoxin A contents in the basal TMR diet were below the detection limits (0.01 µg/kg). AFM_1_ was not detected in the milk samples of all cows during the 7 days before the experiment started as well as in the milk of control cows during the entire experimental period. The milk AFM_1_ contents (66, 49 and 56 ng/kg) of AF, AD1 and AD2 treatments exceeded or were at risk of exceeding the MRL of the European Union (0.05 µg/L). A previous study on lactating dairy cows reported that the plasma AFM_1_ was detectable at 5 min (10.4 ng/L) and peaked at 25 min (136.3 ng/L) after a single oral intake of 4.9 mg AFB_1_ [54]. Furthermore, the study of Frobish et al. disclosed that AFM_1_ appeared in the milk within 12 h after dairy cows receiving AFB_1_ contaminated feed [55]. A plateau of AFM_1_ concentration in the milk was observed on day 7 after AFB_1_ administration and the steady-state condition was maintained up to the last day of the AFB_1_-dosing period (day 14). Likewise, previous studies have confirmed that the plateau of AFM_1_ concentration in the milk was observed at day 1 [33,55] to day 4 [17] after AFB_1_ administration with or without adsorbents in the diet. The present study was consistent with previous results [2,33,43], a sharp decrease of AFM_1_ level in milk was detected within 24 h after withdrawal AFB_1_. The difference was the milk AFM_1_ concentrations (43, 41 and 33 ng/kg) of AF, AD1 and AD2 treatments dropped below the MRL of the European Union. The milk AFM_1_ content continued decreasing and was ultimately undetectable by 5 days after stop administrating AFB_1_. This was consistent with previous studies that the duration of AFM_1_ clearance in the milk of dairy cows could be 3 [2,33,43] to 4 days [17] after the last critical risk (>20 µg/kg) AFB_1_ administration. These findings suggested that moderate risk AFB_1_ (8 µg/kg) administration has a similar effect tendency to AFM_1_ content in milk of apparently healthy dairy cows with a critical level (>20 µg/kg) AFB_1_ administration. The average AFM_1_ concentration in milk at steady state was 93 ng/L in the AF treatment, which was below the AFM_1_ MRL set by the United States and China, but it was 1.86 times higher than the legal limit of the European Union.

The transfer rate of dietary AFB_1_ into milk AFM_1_ is highly correlated with milk yield, the transfer rate usually is 1–2% for late lactating dairy cows (yield < 30 kg/d) and up to 6% for high-yielding cows (yield > 30 kg/d) [56]. Furthermore, the cow species difference, general health, hepatic biotransformation capacity, rate of ingestion and the integrity of the mammary alveolar cell membranes have been shown to affect the transfer rate [56]. According to the transfer rate equation above, 5–10 µg/kg AFB_1_ in the diet of lactating cows converted to 40–430 ng/L AFM_1_ in raw milk, which is below the AFM_1_ MRL set by the United States and China, but at risk of exceeding the legal limit of the European Union. However, the feedstuffs for dairy cows are normally co-exposure to the mycotoxin combinations, due to the possible additive or synergic effect, which may lead to more adverse effects than purified AFB_1_. Thus, the dietary AFB_1_ risk of dairy cows was defined into four levels: critical (>20 µg/kg), high (10–20 µg/kg), moderate (5–10 µg/kg) and low (<5 µg/kg).

The transfer rates of AFB_1_ from the diet into AFM_1_ in milk were 1.16, 0.57 and 0.63% in the AF, AD1 and AD2 treatments. In accordance with the results of our study, the reports of Guo et al. [17], Maki et al. [37] and Ogunade et al. [33] showed the carry-over rates were 1.06%, 1.13% and 1.07% when cows were challenged with critical AFB_1_ dosing of 63 µg/kg, 75 µg/kg and 100 µg/kg, respectively. The highest transfer rate of 7.26% was observed in the report of Malinee et al., while the cows (milk yield of 10 kg per day) were fed with TMR diet contained 22.28 µg/kg AFB_1_ [32]. Furthermore, the inclusion of adsorbents in the AFB_1_ contaminated diet significantly reduced the transfer rates in previous studies [19,37,38,41,42,46] regardless of milk production and dietary AFB_1_ dosage variables (Table 4). In addition to adsorbents, biodegradation products such as *Bacillus subtilis* ANSB060 [17], *Kluyveromyces marxianus* and *Pichia kudriavzevii* which isolated from the ruminal fluid of dairy cows [32] also observably reduced the milk AFM_1_ content and transfer rates. Masoero et al. proposed a linear regression equation to describe the relationship between the carry-over rate of diet AFB_1_ to milk AFM_1_ and the milk yield as follows: carry-over% = −0.326 + 0.077 × milk yield; r^2^ = 0. 58) [57]. Our current data fitted the equation well, with the actual and estimated carry-over rate of 1.16% and 1.28%, respectively. Compared to the AF, the AD1 and AD2 significantly decreased the mean AFM_1_ concentration (93 ng/L vs. 46 ng/L vs. 51 ng/L), AFM_1_ excretion (1.94 μg/d vs. 0.96 μg/d vs. 1.06 μg/d) and the transfer rate (1.16% vs. 0.57% vs. 0.63%), respectively. Although the mean milk AFM_1_ concentrations of AD1 and AD2 treatments significantly decreased to 46 and 51 ng/L, which were below the AFM_1_ MRL set by the United States and China, still at risk of exceeding the legal limit of the European Union.

To assess the risk of AFM_1_ exposure in Chinese populations due to milk AFM_1_ intake under moderate risk level AFB_1_ and adsorbents in the diet of apparently healthy lactating cows, we calculated the estimated daily intake (EDI) and the hazard index (HI) values in different human age groups. Kuiper-Goodman [23] determined a No Observed Effect Level (NOEL) for AFM_1_ of <2.5 g/kg bw/day and proposed the AFM_1_ tolerable daily intake (TDI) of 0.20 ng/kg body weight/day as a “safe dose”, i.e., 50% of the animals would have developed tumors (TD50) dividing by a large safety factor of 50,000. HI above 1.00 indicates that milk AFM_1_ intake is considered a potential risk for liver cancer in consumers [58]. In this study, the EDI values ranged from 0.17 to 1.02, 0.08 to 0.51 and 0.09 to 0.56 ng/kg bw/day, with the HI values, ranged from 0.84 to 5.11, 0.42 to 2.53 and 0.46 to 2.80 in AF, AD1 and AD2, respectively. The HI values of the youth population aged 2–18 years old and the elderly population aged >60 years old in AF were above 1.00, indicates that milk AFM_1_ intake is a potential risk for liver cancer in the public, expressly for youth and elderly consumers. Compared to AF, both AD1 and AD2 had obvious reductions in the HI values in all age groups, which proves that adding adsorbents in the diet of cows is an effective measure to remit milk AFM_1_ exposure risk for humans. However, the HI values of youth consumers aged 2–11 years old were still above 1.00, indicates that adding adsorbents is not a guaranteed measure to eliminate milk AFM_1_ residue, which is still presenting exposure risk for youth consumers aged 2–11 years old. Previous epidemiological surveys have also assessed people’s risk of exposure to AFM_1_ in milk and found that the EDI was 0.242 ng/kg bw/day in Iran [59], was 0.025–0.328 ng/kg bw/day in Italy [60], 0.495 ng/kg bw/day in Lebanon [22], 0.22 ng/kg bw/day [11] and 0.263 ng/kg bw/day [20] in China. Furthermore, the risk of AFM_1_ exposure was highest in milk consumers aged 2–4 years old, with an EDI of 1.02, 0.51 and 0.56 ng/kg bw/day and a HI of 5.11, 2.53 and 2.80 in AF, AD1 and AD2, respectively. These were lower than the EDI of 3.7 ng AFM_1_/kg bw/day for a four-month-old infant weighing 6 kg, representing a daily intake of 22 ng of AFM_1_ reported by Oliveira et al. in Brazil [61]. Peng and Chen conducted a Monte Carlo simulation to estimate the AFM_1_ intake of different population groups in Taiwan and found a mean AFM_1_ intake of 3.25 ng/day for 19 to 44 years old women to 5.67 ng/day for 19 to 44 years old men [62]. Meanwhile, the lowest exposure risk was observed in the population aged 30–45 years old, with the EDI of 0.17 and the HI of 0.84 and increased gradually in people aged above 45 years old. The EDI values were 0.21 and 0.25 ng/kg bw/day and HI values were 1.04 and 1.24 for the elderly population aged 60–70 and >70 years old, respectively. The elderly population may also be sensitive to the adverse effects of AFM_1_ due to decreased immunity and poor physical condition.

According to the TDI of 0.20 ng/kg bw/day and based on the body weight and milk consumption of children aged 2–4 years old in this study, the maximum average concentration of AFM_1_ in milk consumed by these children was calculated to be 18.2 ng/L [11], which is below the AFM_1_ MRL set by the United States, China and the European Union. According to the carry-over equation proposed by Britzi et al. [56], moderate risk level (5–10 µg/kg) AFB_1_ in the diet of apparently healthy lactating cows converted to 40–430 ng/L AFM_1_ in raw milk, posing a significant human health hazard, expressly for youth and elderly population. To our knowledge, this is the first study to assess the human risk of exposure to milk AFM_1_ from cows fed with moderate risk level AFB_1_ and adsorbents. Our results suggested that the inclusion of mycotoxin adsorbents in the dairy diet could decrease AFM_1_ residual in raw milk and reduce the exposure risk for the public.

## 4. Conclusions

Supplemental moderate risk level (8 µg/kg) AFB_1_ and adsorbents in the diet of healthy lactating cows did not affect the behaviors, dry matter intake (DMI), milk yield, milk compositions and serum parameters of the dairy cows. Moderate risk level AFB_1_ significantly increased the AFM_1_ residual in raw milk and the transfer rates of AFB_1_ from the diet into AFM_1_ in milk of apparently healthy cows, posing a significant human health hazard, expressly for the youth and elderly population. The inclusion of mycotoxin adsorbents in the AFB_1_ contaminated diet proved to be an effective measure to remit milk AFM_1_ residue and its exposure risk for humans.

## 5. Materials and Methods

All cow feeding and management in this study were performed according to the China Agricultural University animal research committee protocol (Protocol number: 2013-5-LZ) and all the protocols in present study were approved by the Ethical Committee of China Agricultural University (Protocol number: CAU20180825-2; Date: 25 August 2018).

### 5.1. Experimental Design, Diets and Cow Management

Forty healthy lactating multiparous Holstein cows (parity (mean ± SD) = 3.1 ± 0.3, days in milk = 270 ± 22 d, daily milk yield= 21 ± 3.1 kg/d, bodyweight = 650 ± 25 kg) from the Aomei dairy farm (Xinxiang, Henan province, China) were randomly assigned into one of four treatments: (1) control diet (CON), basal total mixed ration (TMR) without AFB_1_ and adsorbents; (2) aflatoxin diet (AF), CON diet + 168 µg/d AFB_1_ (resulted in 8 µg/kg AFB_1_ of diet dry matter); (3) adsorbent 1 diet (AD1), AF diet + 15 g/d adsorbent 1 (0.07% of diet dry matter); (4) adsorbent 2 diet (AD2), AF diet + 15 g/d adsorbent 2 (0.07% of diet dry matter). The experiment lasted for 19 days, AFB_1_-dosing for 14 days as the AFB_1_-challenge period (day 1 to 14), then following as the AFB_1_-withdraw period (day 15 to 19). Diet was formulated to meet NRC requirements of a dairy cow producing 21 kg/d milk [63]. The ingredients and chemical compositions of the diet are present in Table 6. Adsorbent 1 (Patent ID: CN111296722A, a patented product developed by our laboratory) consists of montmorillonite and diatomite in a ratio of 50:50; adsorbent 2 is a commercial product that consists of montmorillonite, diatomite, yeast cell wall extracts and sodium alginate. Before the trial, we continually measured the daily DMI for 7 days and then calculated the average AFB_1_ intake of cows according to the average DMI. The DMI and AFB_1_ intakes of cows were 21 kg/d and 8 µg/kg/d, respectively. Pure AFB_1_ (purity: 99.5%, Shanghai Yuduo Biotechnology Co., Ltd., Shanghai, China) was dissolved in methanol. The AFB_1_ was administrated daily to each cow in the treatment groups by top-dressing and the adsorbent was manually mixed with TMR. All of the cows in the four treatments were only fed the basal TMR during the AFB_1_-withdraw period. Cows were fed twice daily (07:00 and 17:00). All cows were access to feed and water ad libitum. Two experienced veterinarians assessed and recorded the health condition of the dairy cows every day during the entire trial period.

### 5.2. Sample Collection and Analysis

Samples for TMR in each group were collected and stored at −20 °C. The TMR samples were dried at 65 °C for 48 h in a forced-air oven and ground to pass through a 1 mm screen using a feedstuff mill (KRT-34; KunJie, Beijing, China) subsequently. In addition, the samples were then divided into two portions and stored at −20 °C until the analysis of chemical composition and mycotoxins. The DM, CP of TMR samples were determined according to the methods described by the Association of Official Analytical Chemists (AOAC) [64]. The content of NDF and ADF were analyzed by the Ankom fiber analyzer (A2000i; Ankom Technology, Fairport, NY, USA) following the procedures of Van Soest et al. [65]. The quantification for mycotoxins (AFB_1_, AFB_2_, AFG_1_, AFG_2_, deoxynivalenol, T-2 toxin, zearalenone and ochratoxin A) in diet were determined as previously described by Li et al. [66]. The aflatoxin B_1_, B_2_, G_1_, G_2_, deoxynivalenol, T-2 toxin, zearalenone and ochratoxin A contents in the experimental diets were below the detection limits (0.01 µg/kg).

The cows were milked twice daily (06:30 and 16:30) using a DeLaval milking system and milk yield was recorded at each milking time. Milk samples were collected at each milking time on days 0, 1, 7, 14, 15, 18 and 19 and approximately 100 mL of milk was collected into two 50 mL tubes. Milk samples from one tube were sent to Henan Dairy Herd Improvement (DHI) Testing Center (Zhengzhou, China) for the analysis of milk fat, protein, lactose and somatic cell count (SCC) by an automated near-infrared milk analyzer (Seris300 CombiFOSS; Foss Electric, Hillerød, Denmark). Milk from another tube was stored at −20 °C for mycotoxins analysis. The quantification of AFM_1_ in milk samples was conducted by the Romer Laboratory (Wuxi, China) following the LC-MS/MS method from the Ministry of Health, China [67].

Blood samples were collected from the coccygeal vein before the morning feeding on days 7 and 14 and centrifuged at 3000 rpm for 15 min at 4 °C to obtain the serum. All serum samples were submitted to Huaying Biotechnology Co., Ltd. (Beijing, China). The glucose (Glu), total protein (TP), alanine aminotransferase (ALT), aspartate aminotransferase (AST), nonestesterified fatty acid (NEFA), β-hydroxybutyric acid (BHBA), malondialdehyde (MDA), glutathione peroxidase (GSHPx), total antioxidant capacity (T-AOC), superoxide dismutase (SOD) and total bilirubin (TBIL) were analyzed using Hitachi 7160 automatic biochemical analyzer (Hitachi 7160; Hitachi Incorporated, Tokyo, Japan) through a colorimetric kit (DiaSys Diagnostics Systems GmbH, Frankfurt, Germany). Serum diamine oxidase (DAO), D-lactic acid (D-LA), Lipopolysaccharide (LPS) concentrations were detected using an enzyme-labeled instrument (Thermo Multiskan Ascent, American) with enzyme-linked immunosorbent assay (ELISA).

### 5.3. Risk Assessment of Exposure to AFM_1_

The estimated daily intake (EDI) and the hazard index (HI) of the average AFM_1_ concentration in milk during the platform in the current study were calculated according to the equations as follows:

$$\mathrm{EDI}\;(\mathrm{ng}/\mathrm{kg}\;\mathrm{bw}/\mathrm{day})\;=\;\frac{\left({\mathrm{AFM}}_{1}\;\mathrm{concentration}\;\mathrm{in}\;\mathrm{milk}\right)\;\times \;(\mathrm{daily}\;\mathrm{milk}\;\mathrm{consumption})}{\mathrm{average}\;\mathrm{body}\;\mathrm{weight}}$$

where data on daily milk consumption and average body weight of different ages in China were found in previous studies [11,50,59] and the AFM_1_ contents in the milk of dairy cows fed different diets in the current study were used as the AFM_1_ concentration in milk in this equation.

$$\mathrm{HI}\;=\;\frac{\mathrm{estimated}\;\mathrm{daily}\;\mathrm{intake}\;(\mathrm{EDI})}{\mathrm{tolerable}\;\mathrm{daily}\;\mathrm{intake}\;(\mathrm{TDI})}$$

where TDI as the safe dose, was set as 0.20 ng/kg bw/day as suggested by Kuiper-Goodman (1990), it was determined by dividing the TD50 (the dose at which 50% of the animals would have developed tumors) by a safety factor of 50,000 [23]. A HI value higher than 1 indicates that milk AFM_1_ intake is considered a potential risk for liver cancer in consumers [58].

### 5.4. Calculations

3.5% FCM yield = 0.4324 × milk yield + 16.218 × milk fat yield [63].

AFM_1_ excretion (μg/d) = concentration of AFM_1_ in milk (μg/kg) × milk yield (kg/d).

Transfer rate (%) = excretion of AFM_1_(μg/d)/AFB_1_ consumption (μg/d) × 100%.

### 5.5. Statistical Analysis

The milk yield, milk components, serum parameters and the AFM_1_ content in milk were analyzed by one-way analysis of variance (ANOVA) using SAS (version 9.2; SAS Institute, Cary, NC, USA). A significant difference among the treatments was determined by Duncan’s multiple range tests. The significance level was set at 0.05.

## Figures and Tables

**Table 1:** Effect of the moderate risk level of AFB_1_ with or without adsorbents on the performance of cows at steady state (days 7 to 14) (*n* = 10).

Item ^1^	Dietary Treatment ^2^				SEM	*p*-Value
	CON	AF	AD1	AD2		
DMI (kg/d)	20.88	20.73	20.86	20.82	1.84	0.65
Milk yield (kg/d)	20.85	20.91	20.97	20.82	0.25	0.15
3.5% FCM (kg/d)	23.79	25.13	24.11	25.79	0.71	0.98
Fat (%)	4.87	4.88	4.78	4.85	0.09	0.98
Protein (%)	4.20	4.36	4.22	4.16	0.06	0.68
Lactose (%)	4.81	4.70	4.77	4.81	0.02	0.25
Solid (%)	14.36	14.47	14.26	14.26	0.15	0.96
SCC (× 1000/mL)	196.20	188.50	249.65	190.75	20.52	0.73

^1^ DMI: dry matter intake; 3.5% FCM (kg/d) = 0.432 × milk yield + 16.23 × fat yield; SCC: somatic cell count. SEM: standard error of the mean. ^2^ CON: the basal diet without AFB_1_ and adsorbents; AF: CON + 8 μg/kg AFB_1_; AD1: AF + 15 g/d adsorbent 1; AD2: AF + 15 g/d adsorbent 2.

**Table 2:** Effect of the moderate risk level of AFB_1_ with or without adsorbents on serum metabolite parameters of dairy cows (*n* = 10).

Item ^1^	Dietary Treatment ^2^				SEM	*p*-Value
	CON	AF	AD1	AD2		
Energy metabolism						
GLU (mmol/L)	4.15	3.95	4.33	4.40	0.06	0.31
NEFA (μmol/L)	73.03	60.52	69.68	73.03	3.49	0.19
BHBA (mmol/L)	0.62	0.41	0.48	0.59	0.05	0.12
Liver function						
ALT (U/L)	21.80	26.16	25.76	22.01	0.57	0.12
AST (U/L)	54.34	59.81	61.82	54.07	1.09	0.70
TP (g/L)	73.13	72.42	74.14	75.54	0.60	0.53
Oxidative stress						
T-AOC (U/mL)	9.49	10.45	8.84	8.64	0.37	0.61
GSHPx (μmol/L)	385.86	409.89	402.73	401.43	9.60	0.29
SOD (U/mL)	40.46	37.85	41.51	41.14	1.02	0.99
MDA (nmol/mL)	3.13	2.77	3.13	3.17	0.19	0.58
Gastrointestinal permeability						
DAO (ng/mL)	5.37	4.52	3.99	4.68	0.31	0.56
D-LA (μmol/mL)	16.59	13.34	13.97	15.83	1.13	0.18
LPS (EU/L)	352.72	311.95	331.15	348.74	23.25	0.99

^1^ GLU, Glucose; NEFA, non-esterified fatty acid; BHBA, β-hydroxybutyric acid; ALT, alanine aminotransferase; AST, aspartate aminotransferase; TP, total protein; TAOC, total antioxidant capacity; SOD, superoxide dismutase; GSHPx, glutathione peroxidase; DAO, diamine oxidase; D-LA, D-lactic acid; LPS, Lipopolysaccharide. SEM: standard error of the mean. ^2^ CON: the basal diet without AFB_1_ and adsorbents; AF: CON + 8 μg/kg AFB_1_; AD1: AF + 15 g/d adsorbent 1; AD2: AF + 15 g/d adsorbent 2.

**Table 3:** Effect of the moderate risk level of AFB_1_ with or without adsorbents on the concentration, excretion and transfer rate of AFM_1_ in the milk of dairy cows at steady state (day 7 to 14) (*n* = 10).

Item	Dietary Treatments ^1^				SEM	*p*-Value
	CON	AF	AD1	AD2		
AFB_1_ intake (μg/d)	ND	168	168	168		
AFM_1_ concentration in milk (ng/L)	ND	93 ^a^	46 ^b^	51 ^b^	7	0.04
AFM_1_ excretion ^2^ (μg/d)	ND	1.94 ^a^	0.96 ^b^	1.06 ^b^	0.14	<0.01
Transfer rate ^3^ (%)	\	1.16 ^a^	0.57 ^b^	0.63 ^b^	0.01	<0.01

^a,b^ Values in the same row with no common superscript differ significantly(*p* < 0.05). ^1^ CON: the basal diet without AFB_1_ and adsorbents; AF: CON + 8 μg/kg AFB_1_; AD1: AF + 15 g/d adsorbent 1; AD2: AF + 15 g/d adsorbent 2. ^2^ AFM_1_ excretion (μg/d) = concentration of AFM_1_ in milk (μg/L) × milk yield (kg/d). ^3^ Transfer rate (%) = excretion of AFM_1_ (μg/d)/AFB_1_ consumption (μg/d) × 100. SEM: standard error of the mean.

**Table 4:** Comparison of AFM_1_ transfer rate under different risk levels of dietary AFB_1_ with or without adsorbents in previous studies and the present study.

Study	DIM ^1^ (days)	AFB_1_ Source ^2^	AFB_1_ Dosage (μg/kg)	Milk Yield (kg/day)	Detoxification Agent	Agent Dosage (%) ^3^	Transfer Rate (%)
Maki et al., 2016 [37]	114 ± 14	*Ap* (NRRL-2999) culture (758 mg/kg)	117	21.30	\	\	1.07
				21.20	NovaSil Plus	0.5%	0.52
				20.60	NovaSil Plus	1%	0.32
Kutz et al., 2009 [38]	163 ± 54	*Ap* (NRRL-2999) culture (760 mg/kg)	112.2	34.19	\	\	2.65
				34.13	Solis	0.56%	1.48
				33.73	NovasilPlus	0.56%	1.42
				34.43	MTB-100	0.56%	2.52
Weatherly et al., 2018 [39]	153 ± 83	*Ap* (NRRL-2999) culture (102 mg/kg)	100	32.3	\	\	\
				35.0	adsorbent	30 g/day	\
				32.1	adsorbent	60 g/day	\
				33.7	PROT	60 g/day	\
Pate et al., 2018 [40]	157 ± 43	*Ap* (NRRL-2999) culture (102 mg/kg)	100	35.59	\	\	0.45
				38.14	FloMatrix	113 g/day	0.49
				37.17	FloMatrix	227 g/day	0.39
Sulzberger et al., 2017 [41]	146 ± 69	*Ap* (NRRL-2999) culture (102 mg/kg)	100	37.83	\	\	1.37
				37.57	Clay	0.5%	1.01
				37.28	Clay	1%	0.98
				36.44	Clay	2%	0.74
Rodrigues et al., 2019 [42]	183 ± 70	*Ap* (NRRL-2999) culture (650 mg/kg)	76.87	37.1	\	\	2.70
			77.65	36.1	Toxy-Nil	0.4%	1.00
			73.97	37.8	Unike Plus	0.4%	1.30
Ogunade et al., 2016 [33]	150–200	*Ap* (NRRL-2999) culture (Not described)	75	26.6	\	\	1.13
				26.5	SEQ1	20 g/day	1.14
				26.7	SEQ2	20 g/day	1.11
				26.1	SEQ3	20 g/day	1.08
Queiroz et al., 2012 [43]	295 ± 45	*Ap* (NRRL-2999) culture (640 mg/kg)	75	18.9	\	\	0.61
				19.9	Calibrin A	0.2%	0.75
				19.1	Calibrin A	1%	0.51
Guo et al., 2019 [17]	254 ± 19	Pure AFB_1_	63	20	\	\	1.06
			64	20	BDP (ANSB060)	0.2%	0.76
Maki et al., 2017 [44]	Not described	*Ap* (NRRL 2999) culture (758 mg/kg)	50	36.45	\	\	1.78
				36.27	Novasil Plus	0.125%	1.50
				36.18	Novasil Plus	0.25%	1.46
Xiong et al., 2015 [2]	271 ± 29	*Af* (No. 3.4409) culture (28.8 mg/kg)	20	21.3	\	\	0.56
				21.3	Solis Mos	0.25%	0.46
			40	22.4	\	\	0.59
				22.6	Solis Mos	0.25%	0.57
Sumantri et al., 2012 [45]	84–98	Naturally contaminated ground peanut meal (1358 and 13 μg/kg)	0.30	6.75	\	\	0.12
			30.62	6.72	\	\	0.10
			30.81	6.85	Bentonite	0.5%	0.10
			30.65	7.27	Bentonite	2.0%	0.10
Intanoo et al., 2020 [32]	180 ± 21	Naturally contaminated diet (22.28 μg/kg)	22.28	10.03	\	\	7.26
				10.23	CPY1	2 g/day	1.18
				10.18	RSY5	2 g/day	1.44
				10.10	YSY2	2 g/day	1.69
Xiong et al., 2018 [19]	33 ± 7	*Af* (No. 3.4409) culture (28.8 mg/kg)	20	35.7	\	\	1.38
				35.5	Solis Mos	0.25%	0.89
Masoeroa et al., 2009 [46]	120 ± 22	Naturally contaminated corn meal (32.13μg/kg) and Pmx (4.13 μg/kg)	7.31	31.03	\	\	3.80
			7.47	33.25	Cay SA	0.83%	2.10
Polat et al., 2015 [47]	Passed peak	Naturally contaminated diet from 20 dairy farms	5.778	19.9	\	\	2.66
Mojtahedi et al., 2013 [48]	95 ± 17	Naturally contaminated diet (4.6 μg/kg)	4.6	37.8	\		1.30
				37.3	EG	18 g/day	1.47
				37.6	EG	27 g/day	1.86
				37.6	EG	36 g/day	1.24
Costamagna et al., 2019 [49]	<90	Naturally contaminated diet (3.4μg/kg)	3.4	34.12	\	\	0.88
	90–150			30.54	\	\	1.09
	>150			20.15	\	\	0.56
Present study	270 ± 22	Pure AFB_1_	8	20.85	\	\	1.16
				20.91	adsorbent 1	15 g/day	0.57
				20.97	adsorbent 2	15 g/day	0.63

^1^ DIM: Days in milk of the cows used in trails. ^2^ *Ap*: *Aspergillus parasiticus*; *Af*: *Aspergillus flavus*; AFB_1_ concentration of the AFB_1_ source. ^3^ % of Diet DM.

**Table 5:** Effect of the moderate risk level of AFB_1_ with or without adsorbents on the estimated daily intake (EDI) and the hazard index (HI) in different human age groups.

Age	Milk Consumption ^1^ (mL/d)	Average Body Weight ^2^ (kg)	EDI ^3^			HI ^4^		
			AF	AD1	AD2	AF	AD1	AD2
2–4	151.7	13.8	1.02	0.51	0.56	5.11	2.53	2.80
4–7	130.2	17.9	0.68	0.33	0.37	3.38	1.67	1.85
7–11	136.8	25.6	0.50	0.25	0.27	2.49	1.23	1.36
11–14	141.0	36.3	0.36	0.18	0.20	1.81	0.89	0.99
14–18	133.8	49.2	0.25	0.13	0.14	1.26	0.63	0.69
18–30	120.5	57.7	0.19	0.10	0.11	0.97	0.48	0.53
30–45	109.0	60.1	0.17	0.08	0.09	0.84	0.42	0.46
45–60	118.9	59.7	0.19	0.09	0.10	0.93	0.46	0.51
60–70	127.2	57.0	0.21	0.10	0.11	1.04	0.51	0.57
>70	142.4	53.6	0.25	0.12	0.14	1.24	0.61	0.68

^1,2^ Data on Milk consumption and Average body weight are from the previous studies [11,50]. ^3^ EDI: estimated daily intake (ng/kg bw/day). Milk AFM_1_ concentrations were 93 ng/L, 46 ng/L and 51 ng/L for AF, AD1 and AD2, respectively, which were used to calculate the EDI values. ^4^ HI: hazard index, which was calculated as follows: HI = EDI/tolerable daily intake (TDI), where TDI was set as 0.20 ng/kg bw/d suggested by Kuiper-Goodman [23].

**Table 6:** Ingredients and chemical compositions of the basal diet.

Ingredients ^1^	Amount (% of DM)
Corn silage	41.72
Alfalfa silage	8.83
Oat hay	4.07
Corn-steam flaked	7.94
Soybean meal	5.96
Ground Corn	12.35
Wheat bran	1.99
Cottonseed meal	7.62
Extruded soybean	1.53
DDGS	2.8
Bicarb	1.48
Premix ^2^	2.87
Magnesium oxide	0.48
Yeast	0.36
Chemical levels (% of DM)	
CP	16.03
EE	3.04
NDF	31.85
ADF	18.5
Ash	8.16
NE_L_^3^ (MJ/kg)	1.59
Ca (g/kg)	0.8
P (g/kg)	0.4
Aflatoxin B_1_, B_2_, G_1_, G_2_ (μg/kg)	ND ^4^
Deoxynivalenol (μg/kg)	ND
T-2 toxin (μg/kg)	ND
Zearalenone (μg/kg)	ND
Ochratoxin A (μg/kg)	ND

^1^ DM: dry matter; DDGS: dry distilled grain soluble; CP: crude protein; EE: Ether extract; NDF: neutral detergent fiber; ADF: acid detergent fiber; NE_L_: net energy for lactation. ^2^ Premix was Formulated with 20% salt, 18% Ca, 10% P, 800 mg/kg Cu, 700 mg/kg Mn, 800 mg/kg Zn, 20 mg/kg Fe, 125 mg/kg I, 80 mg/kg Se; 70 mg/kg Co, 300,000 IU/kg vitamin A, 7600 IU/kg vitamin D_3_, 10,000 IU/kg Vitamin E. ^3^ NE_L_ was a calculated value, while the others were measured values. ^4^ ND: not detected.

## Data Availability

Data are contained within the article.
